# Pricing air pollution: evidence from short-term exposure to air pollution on hospitalization of acute bronchitis and chronic obstructive pulmonary disease in southwestern China

**DOI:** 10.1093/inthealth/ihab071

**Published:** 2021-11-19

**Authors:** Pei Zhang, Xiaoyuan Zhou

**Affiliations:** West China School of Public Health and West China Fourth Hospital, Sichuan University, Chengdu, 610041, China; West China School of Public Health and West China Fourth Hospital, Sichuan University, Chengdu, 610041, China

**Keywords:** ambient air pollution, cost of illness method (COI), DLNM, economic loses, hospital admissions

## Abstract

Existing evidence suggests that ambient air pollution has serious adverse effects on respiratory diseases, yet there is little direct evidence from China regarding corresponding economic losses. Here we quantified air pollution–related acute health effects and related economic losses of the most common two respiratory diseases in southwestern China, acute bronchitis and chronic obstructive pulmonary disease (COPD). We applied a distributed lag non-linear model to analyse the relationship between ambient air pollutants and hospital admissions of acute bronchitis and COPD, then applied the cost of illness method to explore the attributing economic burden. During the study period, 528 334 and 99 419 hospital admissions of acute bronchitis and COPD, respectively, were recorded. As a result, during the study period the total hospitalization economic losses attributable to air pollution were 486.40 and 254.74 million yuan for acute bronchitis and COPD, respectively, accounting for 0.015% of local gross domestic product. Our research provides intuitive evidence on the health and economic impacts of short-term exposure to air pollution, which is a key basis for the formulation of environmental policies.

## Introduction

Ambient air pollution has become one of the most concerning issues around the world. More than 91% of the population worldwide are living in places where air quality exceeds World Health Organization (WHO) guidelines, resulting in 4.2 million premature deaths each year.^1,2^ In the past few decades, adverse effects of ambient air pollutants on respiratory diseases have been drawing universal attention. Previous studies provided consistent evidence that exposure to air pollutants could augment the burden of respiratory diseases from hospital outpatient visits,^3^ emergency department visits,^4,5^ hospital admissions^6,7^ and mortality.^8,9^ These studies primarily estimated disease burden through disability-adjusted life years (DALYs)^10^ and years of life lost (YLL).^11^

Recently, research in this field has been promoted through the introduction of economic evaluation methods. The most commonly used methods include the value of statistical life (VSL),^12,13^ the human capital approach (HCA)^14,15^ and the cost of illness method (COI).^10,16^ Among these methods, the VSL and HCA are mainly used to assess the economic burden of mortality, while the COI is often used to evaluate the economic burden of morbidity. Because of these methods, health effects of air pollutants can now be measured not only by the risk of disease, but also by economic burden. For instance, a study in Los Angeles suggested that near-roadway nitrogen dioxide (NO_2_) exposure would lead to an external economic loss to society of ${\$}$104 million in 2007.^12^ Another study in Beijing found that the external costs of 2.5 μm particulate matter (PM_2.5_) equalled around 0.3% of regional gross domestic product (GDP) in 2012.^14^

Despite the application of the aforementioned new methods, however, the economic impact of air pollutants on respiratory diseases still has not been fully explored. Existing studies around the world largely focus on long-term exposure when assessing the economic burden associated with air pollution, without adequate consideration for short-term exposure.^17^ In addition, as non-fatal diseases, although respiratory diseases may cause patients more morbidity than mortality, most scientific evidence from existing research refers to the economic burden of mortality rather than morbidity.^17–19^ Furthermore, only a limited number of studies have focused on measurement of the economic burden of respiratory diseases in underdeveloped areas, although people in these areas are more seriously harmed by air pollutants than their counterparts in developed areas.^1,20^

This study aimed to quantify the association between short-term exposure to air pollutants (NO_2_, ozone [O_3_], PM_10_, PM_2.5_ and sulphur dioxide [SO_2_]) and hospital admissions of acute bronchitis and chronic obstructive pulmonary disease (COPD) and the corresponding hospitalization economic burden of these two diseases caused by air pollutants in southwestern China. To our knowledge, this is the first attempt to evaluate the hospitalization economic burden attributable to short-term exposure to air pollution in China. The results of this study may provide a scientific basis for policymakers to formulate and optimize environmental policies.

## Methods

### Study area and population

Chengdu, located in southwestern China (latitude 30°05′–31°26′ N and longitude 102°54′–104°53′ E), is the capital city of Sichuan Province and the largest city in southwestern China. With the rapid development of industrialization and urbanization, the local air pollution problem is becoming increasingly severe.^21^ The large population, unfavourable atmospheric diffusion conditions and relatively high humidity have turned it into one of the most polluted cities in the country.^22,23^ Our study involved almost all acute bronchitis and COPD hospital admissions cases in Chengdu, guaranteeing enough statistical power to reveal the association between air pollution and hospitalization for these two diseases.

### Data collection

#### Hospitalization data

Official records of hospital admissions for acute bronchitis (International Classification of Diseases, Tenth Revision [ICD-10] code: J20.904) and COPD (ICD-10 code: J44.901) between 1 January 2013 and 15 October 2017 (1749 days in total) were obtained from the local Compulsory Medical Insurance Database. These hospital admissions data came from 1161 hospitals where the ICD-10 was used to classify diagnoses. During the study period, 528 334 hospital admissions for acute bronchitis and 99 419 for COPD were recorded. In order to keep the patients information safe, the identification numbers and names of the patients were separated from the admissions and claims data, which were only manipulated for modelling.

#### Pollution data

Daily average concentrations of NO_2_, PM_10_, PM_2.5_ and SO_2_ and the daily maximum 8-h average concentration of O_3_ were collected from the Sichuan Environmental Monitoring Center. Since our study included almost all acute bronchitis and COPD hospital admissions cases in Chengdu, we obtained the ambient concentration data by averaging the daily mean values of the 12 environmental monitoring stations in the study area.

#### Meteorological data

To control the confounding factors, we also collected the meteorological data. Data on daily average temperature (in °C) and relative humidity (in %) during the study period were obtained from the China Meteorological Data Sharing Service System.

### Modelling

To estimate the health and economic impact of hospitalization attributed to ambient air pollution, we conducted our statistical analysis in three steps: estimating the exposure–response function, calculating the total number of air pollution–related hospital admissions and evaluating the corresponding hospitalization economic burden.

#### Estimating the exposure–response function

In our model, quasi-Poisson regression was used as the linked function because daily hospital admissions typically followed an overdispersed Poisson distribution. Natural spline functions were used to control for the influence of temperature, relative humidity and long-term trends. The model formula is as follows:

(1) \begin{eqnarray*} &&{Y} \sim {quasiPoisson }(\mu)\nonumber\\ &&\mu = \alpha + w_{x,t} \eta + NS(Temp,d{f_{1}}) + NS(RH,d{f_{2}})\nonumber\\ &&\qquad+\, {NS(Time,d{f_{3}}) + Dow} , \end{eqnarray*}

where *Y* is daily hospital admissions, μ is the estimate of *Y*, α is the intercept and *w_x,t_*η is the cross-basis of an exposure–lag response bidimensional function. We used a natural cubic spline function to estimate the lag effects and a linear function to mimic the exposure–response patterns of air pollutants and hospital admissions.^24,25^ NS refers to the natural spline function; df is the degrees of freedom; Temp and RH are daily mean temperature and relative humidity, respectively; Time is included to control long-term temporal trends and Dow is the day of the week, which can be set as a dummy variable to control the systematic variation within a week. According to previous studies,^3^,^26–28^ we chose 7 d as the maximum lag for air pollutants, used 3 as the degrees of freedom of temperature and relative humidity and set the degrees of freedom of the date variable to 7 per year.

After analysing the overall effects, we explored whether the effects of air pollutants on hospital admissions were modified by gender, age and season. The results were illustrated as percentage changes of hospital admissions for a 10 μg/m^3^ increase in air pollutant concentrations.

To test the robustness of our model, we examined the potential influence of df by changing the df value for the date variable from 6 to 8 and changing the df value for temperature from 2 to 4 (the results of the sensitivity analysis are provided in the Supplementary Materials, Table S1). All analyses were completed in R version 3.5.3 (R Foundation for Statistical Computing, Vienna, Austria), using the DLNM package. Statistical tests were two-tailed and a p-value <0.05 was considered statistically significant.

#### Calculating the total number of air pollution–related hospital admissions

Based on the exposure–response coefficients obtained by DLNM, we further calculated the number of air pollution–related hospital admissions of acute bronchitis and COPD. Given the fact that existing research has not yet established a threshold that would not have a detrimental effect on health,^2^^,29^,^30^ we set the reference concentration of all air pollutants to 0 μg/m^3^ in this part of the analysis. The formula is as follows:

(2) \begin{eqnarray*} \begin{array}{@{}*{1}{l}@{}} {R{R_{ij}} = {e^{{\beta _j}\left( {{x_{ij}} - {x_0}} \right)}}}\\ {Percentage\,chang{e_{ij}} = R{R_{ij}} - 1}\\ {A{R_{ij}} = \displaystyle\left( {\frac{{\left( {R{R_{ij}} - 1} \right)}}{{R{R_{ij}}}}} \right) = \frac{{{e^{{\beta _j}\left( {{x_{ij}} - {x_0}} \right)}} - 1}}{{{e^{{\beta _j}\left( {{x_{ij}} - {x_0}} \right)}}}}}\\ {{\rm{\Delta }}n = \sum\limits_{j = 1}^4 {\sum\limits_{{\rm{i = 1}}}^{{\rm{1749}}} {(A{R_{ij}} \times {n_i})} } } \end{array}, \end{eqnarray*}

where *i* represents the day number of the study period (from 1 to 1749); *j* represents air pollutants NO_2_, PM_10_, PM_2.5_ and SO_2_, which have been shown to be harmful to the respiratory system in the first analysis stage; *AR_ij_* is attributable risk; β_*j*_ is the exposure–response coefficient obtained from the first stage, which means the change in hospital admissions caused by a unit change of air pollutants concentration, keeping all the other explanatory variables constant; *x_ij_* is the daily concentration of air pollutants; *x*_0_ is the reference concentration; *n_i_* is the number of daily hospital admissions and Δ*n* is the total number of hospital admissions attributable to these four air pollutants during the study period.

#### Evaluating the corresponding hospitalization economic loss

We adopted the COI method^31^ when evaluating the air pollution–related hospitalization economic loss. The COI method took into account both the costs of hospitalization and the costs due to absence of work. Because we could not obtain the income information of each hospital admission, we used the regional daily GDP per capita to calculate the economic costs resulting from absence of work. The economic burden was obtained according to the following calculations:

(3) \begin{eqnarray*} \begin{array}{@{}*{1}{l}@{}} {meanC{\rm{ = }}{C_h}{\rm{ + }}GD{P_P} \times mean{T_h}}\\ {\Delta C = \Delta n \times meanC} \end{array}, \end{eqnarray*}

where *meanC* is the average economic costs per hospital admission, which includes costs due to hospitalization and those attributable to absence of work; *C_h_* is the mean cost per hospital admission; *GDP_P_* is the daily *GDP* per capita; *meanT_h_* is the mean hospitalization days per hospital admission and Δ*C* is the total economic costs due to ambient air pollutants during the 1749 study days.

In China, hospitals are categorized into three levels according to Chinese Hospital Classification Management Standard: primary, secondary and tertiary. Generally, tertiary hospitals are top-quality service. Hospitalization costs differ a lot in different hospital levels. Therefore subgroup analysis of gender, age and hospital level was also conducted.

## Results

**Figure 1.**
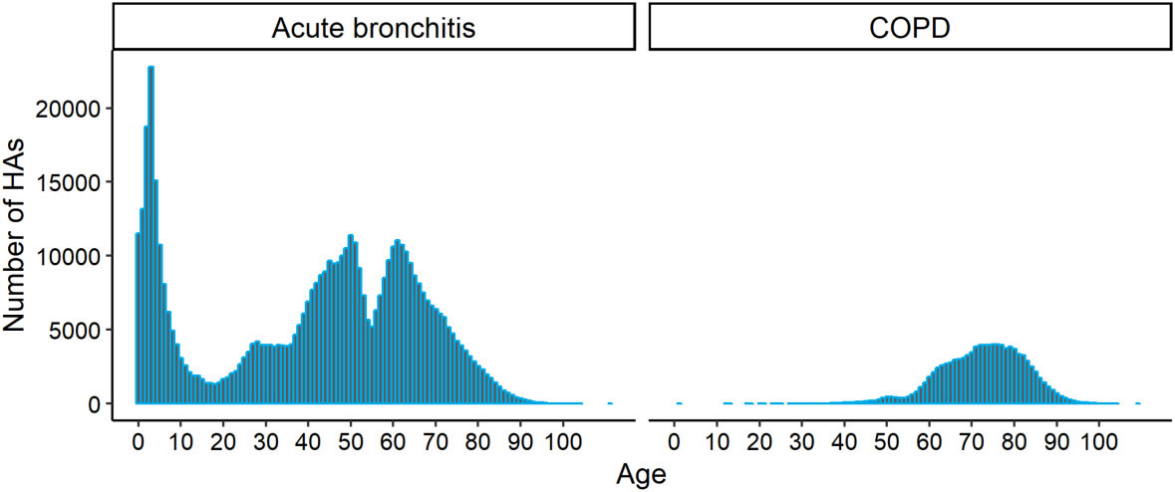
Age distributions of acute bronchitis and COPD hospitalizations. The y-axis represents the daily number of hospital admissions. The x-axis represents different ages.

Descriptive results of daily air pollutant concentrations, meteorological factors and hospital admissions of acute bronchitis and COPD during the study period are summarized in Table 1. Compared with the WHO air quality guidelines of 25 μg/m^3^ and 50 μg/m^3^ for PM_2.5_ and PM_10_, the corresponding average concentrations in Chengdu were 1.8 and 1.3 times higher during the study period. The mean relative humidity was 80.51%, with a standard deviation of 8.57%. The mean temperature was 16.91°C, ranging from −1.9°C to 29.8°C. On each day, the mean number of hospital admissions was 302 and 57 for acute bronchitis and COPD, respectively. Stark differences were observed in age distributions of acute bronchitis and COPD hospitalizations, as shown in Figure 1. Acute bronchitis covered a wider age range and included three peak age groups, while COPD predominantly occurred in the elderly. This finding provided us with clues for a more practical subgroup analysis of age. During the entire study period we defined unequal subgroups of age for these two diseases (age ≤20, 20–55 and ≥55 y for acute bronchitis; age ≤65, 65–80 and ≥80 y for COPD).

**Table 1.** Daily data on air pollutant concentrations, meteorological factors and hospital admissions in Chengdu (2013–2017)

Variables	Mean±SD	Minimum	P_25_	Median	P_75_	Maximum
NO_2_ (μg/m^3^)	55.93±18.32	15.0	43.0	53.0	66.0	144.0
O_3_ (μg/m^3^)	90.25±53.12	4.0	49.0	80.0	129.0	301.0
PM_10_ (μg/m^3^)	114.74±77.96	16.0	61.0	93.0	146.3	862.0
PM_2.5_ (μg/m^3^)	70.88±51.03	6.0	36.0	55.0	90.0	427.0
SO_2_ (μg/m^3^)	18.16±12.3	4.0	11.0	14.0	21.0	96.0
Relative humidity (%)	80.51±8.57	42.0	76.0	81.0	87.0	98.0
Mean temperature (°C)	16.91±7.25	−1.9	10.3	18.0	23.1	29.8
HAs of acute bronchitis	302.08±105.49	49.0	234.0	295.0	360.0	1312.0
Man	127.25±43.17	20.0	101.0	124.0	150.0	541.0
Woman	174.82±64.04	26.0	132.0	169.0	210.0	771.0
Age ≤20 y	77.54±33.82	1.0	56.0	75.0	93.0	508.0
Age >20–<55 y	117.20±41.21	17.0	89.0	113.0	142.0	384.0
Age ≥55 y	107.40±42.59	13.0	78.0	102.0	132.0	420.0
HAs of COPD	56.84±24.92	2.0	42.0	54.0	68.0	385.0
Man	32.45±13.74	1.0	24.0	31.0	39.0	210.0
Woman	24.40±12.29	1.0	17.0	23.0	30.0	175.0
Age ≤65 y	13.10±7.00	1.0	9.0	12.0	16.0	80.0
Age >65–<80 y	29.18±13.47	1.0	21.0	28.0	36.0	209.0
Age ≥80	14.65±6.61	1.0	10.0	14.0	18.0	96.0

P_25_: 25th percentile; P_75_: 75th percentile; HAs: hospital admissions.

**Figure 2.**
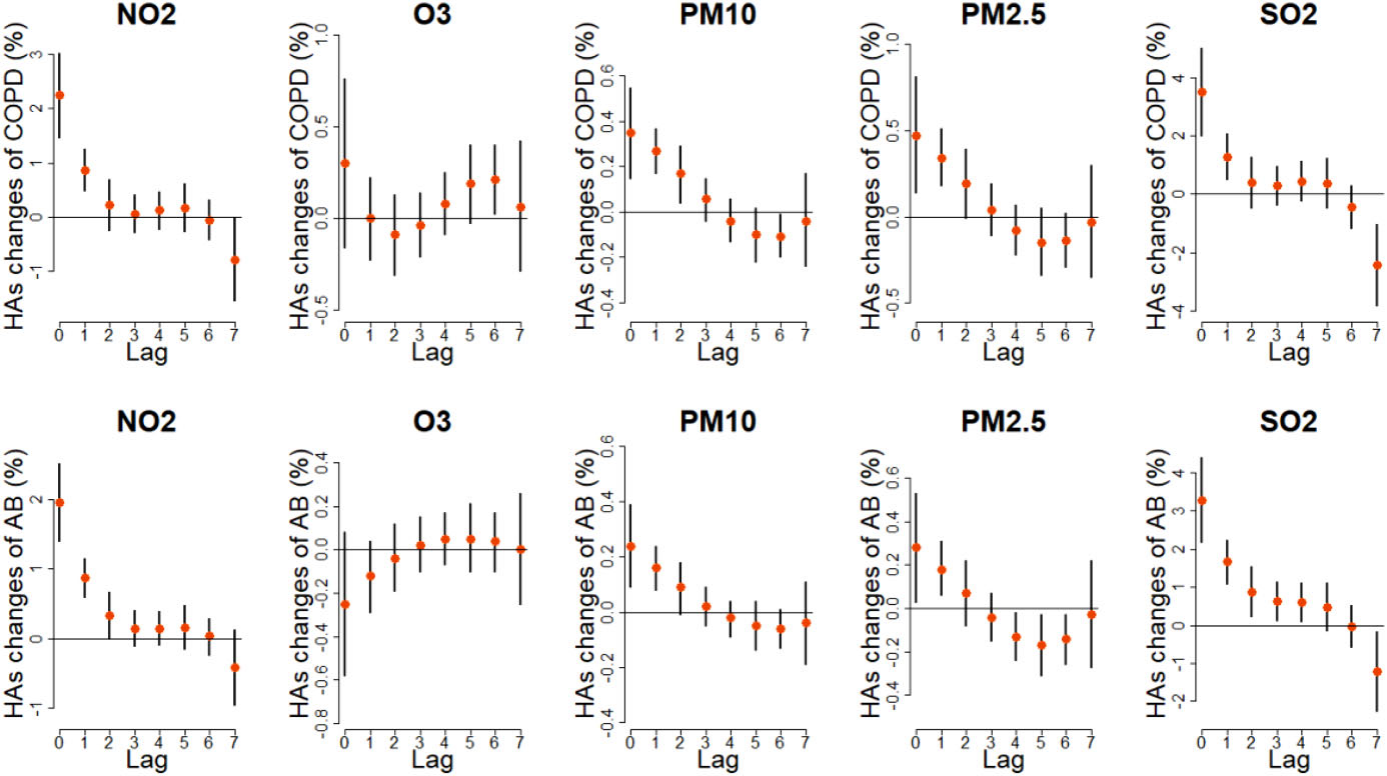
The lag-specific effects of ambient air pollutants on acute bronchitis (AB) and COPD. The y-axis represents daily hospital admissions. The x-axis represents different lag days. The vertical lines represent 95% confidence intervals. The points represent the percent change in hospital admissions.

Figure 2 illustrates the lag-specific relationship of air pollutants and hospital admissions of acute bronchitis and COPD with a 10 μg/m^3^ increase in pollutant concentrations. All pollutants except O_3_ had substantially negative impacts on both acute bronchitis and COPD (p<0.05). They shared a similar lag pattern, with effects most pronounced on lag 0 d and least for 2–3 d. Also, we found the so-called harvest phenomenon easily when analysing the effects of PM_2.5_ on acute bronchitis. Overall cumulative exposure–response associations and three-dimensional graphs of bidimensional exposure–lag response are provided in the Supplementary Materials, Figures S1 and S2, respectively.

Table 2 shows the percentage changes in hospital admissions for different subgroups with a 10 μg/m^3^ increase in pollutant concentrations at lag 0 d. In the subgroup analysis of gender, it was obvious that air pollutants were more harmful to women. In the subgroup analysis of age, the results of these two diseases were quite different. For acute bronchitis, NO_2_ seriously harmed the elderly, while PM_10_, PM_2.5_ and SO_2_ had the greatest influence on people <20 y of age. For COPD, all kinds of air pollutants harmed people ages 65–80 y the most, except SO_2_, where the adverse effect peaked among people ≥80 y of age. O_3_ had no significant influence on hospital admissions for any of the subgroups. The results of subgroup analysis for seasons are also summarized in the Supplementary Materials, Figure S3.

**Table 2.** Percentage changes in hospital admissions for different subgroups with a 10 μg/m^3^ increase in pollutant concentrations

		Percentage change in hospital admissions (95% CI)				
Disease	Group	NO_2_	O_3_	PM_10_	PM_2.5_	SO_2_
Acute bronchitis	Overall	1.95 (1.39 to 2.51)	−0.25 (−0.58 to 0.08)	0.24 (0.09 to 0.39)	0.28 (0.03 to 0.53)	3.28 (2.17,4.41)
	Male	1.69 (1.09 to 2.28)	−0.28 (−0.63 to 0.06)	0.24 (0.07 to 0.40)	0.27 (0.00 to 0.54)	3.08 (1.89 to 4.29)
	Female	2.14 (1.56 to 2.72)	−0.22 (−0.56 to 0.12)	0.24 (0.09 to 0.40)	0.28 (0.02 to 0.54)	3.43 (2.27 to 4.59)
	Age ≤20 y	1.86 (1.07 to 2.65)	−0.24 (−0.70 to 0.22)	0.39 (0.18 to 0.61)	0.49 (0.13 to 0.85)	3.74 (2.12 to 5.38)
	Age ≥20–≤55	1.92 (1.34 to 2.49)	−0.21 (−0.54 to 0.13)	0.21 (0.06 to 0.37)	0.26 (0.00 to 0.51)	3.14 (2.00 to 4.29)
	Age ≥55	2.20 (1.57 to 2.84)	−0.37 (−0.74 to 0.00)	0.18 (0.01 to 0.36)	0.17 (−0.11 to 0.45)	3.43 (2.17 to 4.70)
COPD	Overall	2.24 (1.46 to 3.02)	0.30 (−0.16 to 0.76)	0.35 (0.15 to 0.55)	0.47 (0.14 to 0.81)	3.51 (2.01 to 5.03)
	Male	1.96 (1.14 to 2.80)	0.27 (−0.21 to 0.76)	0.32 (0.10 to 0.54)	0.44 (0.09 to 0.80)	2.91 (1.30 to 4.54)
	Female	2.61 (1.69 to 3.54)	0.34 (−0.21 to 0.89)	0.39 (0.15 to 0.63)	0.51 (0.12 to 0.90)	4.27 (2.52 to 6.05)
	Age ≤65 y	2.16 (1.08 to 3.26)	0.28 (−0.36 to 0.93)	0.22 (−0.07 to 0.51)	0.29 (−0.18 to 0.75)	2.54 (0.50 to 4.61)
	Age ≥65–≤80 y	2.47 (1.60 to 3.35)	0.50 (−0.01 to 1.02)	0.45 (0.22 to 0.68)	0.62 (0.25 to 1.00)	3.76 (2.07 to 5.47)
	Age ≥80 y	1.88 (0.90 to 2.86)	−0.14 (−0.70 to 0.43)	0.26 (0.00 to 0.52)	0.34 (−0.08 to 0.76)	4.03 (2.13 to 5.96)

Table 3 indicates the economic burden caused by NO_2_, PM_2.5__–__10_, PM_2.5_ and SO_2_ from 2013 to 2017. The total economic costs due to air pollutants were approximately 486.40 and 254.74 million yuan for acute bronchitis and COPD, respectively, accounting for 0.015% of local GDP. Most of the air pollutant–related economic burden was attributed to NO_2_ and SO_2_. For acute bronchitis, the pollution-related economic burden of hospitalization mostly comes from people >55 y of age, while for COPD it was people 65–80 y of age.

**Table 3.** Economic costs of acute bronchitis and COPD hospitalizations due to pollutants in subgroups of age and hospital level in Chengdu during the study period

				Economic cost due to pollutants (million yuan)				
		Total number	Hospitalization cost per					
				
Disease	Subgroup	of HAs	capita (yuan)^a^	NO_2_	PM_2.5–10_	PM_2.5_	SO_2_	Total
Acute bronchitis	Tertiary hospitals	61 017	3593.51	29.02	6.83	14.91	14.97	65.73
	Secondary hospitals	121 288	2738.72	63.06	11.78	26.77	30.5	132.11
	Primary hospitals	346 029	1758.35	136.79	20.64	36.05	82.68	276.16
	Age ≤20 y	135 392	1756.97	46.91	14.19	27.24	31.16	119.50
	Age ≥20–≤55 y	205 026	2131.39	86.34	14.72	26.09	48.64	175.79
	Age ≥55 y	187 916	2580.99	105.94	19.55	18.85	55.98	200.32
	Total	528 334	2196.48	239.19	48.46	72.18	135.78	486.40
COPD	Tertiary hospitals	16 419	14 806.52	15.75	2.14	2.08	11.02	30.99
	Secondary hospitals	27 318	7474.46	39.54	13.99	26.34	16.92	96.79
	Primary hospitals	55 682	2902.02	41.63	6.77	16.29	23.63	88.32
	Age ≤65 y	22 826	5422.85	22.92	2.77	6.71	9.36	41.76
	Age ≥65–≤80 y	51 001	5977.16	62.66	19.55	34.33	32.17	148.71
	Age ≥80 y	25 592	7043.70	27.9	5.54	11.03	19.8	64.27
	Total	99 419	6125.31	113.48	27.86	52.07	61.33	254.74

a US${\$}$1 is approximately equal to 6.82 yuan.

HAs: hospital admissions.

## Discussion

In this study we found that short-term exposure to ambient air pollutants may increase hospital admissions of acute bronchitis and COPD after adjusting for temperature and relative humidity. It was also shown that air pollutants caused a heavy economic burden for hospitalization to the society. The results provide updated evidence for policymakers to formulate more effective environmental protection policies.

In the overall analysis, we found positive associations between NO_2_, PM_10_, PM_2.5_ and SO_2_ and hospital admissions of acute bronchitis and COPD. Consistent with existing research in China, the greatest adverse effects of NO_2_, PM_10_, PM_2.5_ and SO_2_ occurred at lag 0 d, followed by a sharp decrease afterwards.^3,24^^,29^ These effects lasted 2–3 d. The results indicate that people were more susceptible to exposures of these pollutants during very short periods between exposure and hospital admission. These adverse effects were explained in previous studies^7,32^ as a result of airway inflammation caused by pollution-induced oxidative stress. Nonetheless, for O_3_, we did not observe any significant adverse effect in the analysis. Although studies in Guangzhou^4^ and Beijing^33^ had results similar to ours, abundant published literature in other countries states that O_3_ is harmful to the respiratory system.^9,34,35^ For example, a study in Greece found that an increase of 10 μg/m^3^ in weekly O_3_ concentration was associated with a decrease in forced vital capacity and forced expiratory volume in 1 s.^36^ Another study in the USA suggested that ozone was associated with paediatric respiratory morbidity in multiple US cities, and the strongest overall association was in Atlanta (odds ratio  1.08 [95% confidence interval {CI} 1.06 to 1.11]).^37^ This discrepancy may result from demographic specificity, which means the susceptibility to air pollutants varies by ethnicity. More relevant epidemiological studies are needed to verify this statement. Also, we found the so-called harvest phenomenon at lag 4–6 d when exploring the effects of PM_2.5_ on acute bronchitis. This may be because acute bronchitis can occur in fragile people immediately after exposure to PM_2.5_, leaving limited numbers of subjects at risk, and the overall long-term hazard was reduced.^38^

The results of subgroup analysis were different. In the subgroup analysis of gender, we found that women suffered more from air pollutants than men for both acute bronchitis and COPD. For example, an increase of 10 μg/m^3^ in NO_2_ resulted in a 2.61% (95% CI 1.69 to 3.54) increase in female COPD hospitalizations compared with 1.96% (95% CI 1.14 to 2.80) in males. This result was in line with previous research.^27^^,39^,^40^ However, a recent study in Chengdu showed that air pollution was more harmful to males, according to hospital admissions data from 2015 to 2016.^41^ Such gender differences were inconsistent with our findings, possibly attributed to differences in study design, sample size and modelling strategies. In the subgroup analysis of age, effects varied with diseases and pollutants. For acute bronchitis, PM_10_, PM_2.5_ and SO_2_ significantly affected people <20 y of age. This finding reconfirmed the existing evidence that air pollution primarily leads to acute bronchitis among infants and children.^42^ Nonetheless, it should be noted that the hazards of NO_2_ were considerably connected with people >55 y of age. Because few studies are related to acute bronchitis, and most of them have concentrated on younger age groups, we need more relevant evidence to support this result. As can be seen from Figure 2, people ≥20 y of age account for more than two-thirds of all acute bronchitis hospitalizations, indicating more attention should be paid to this age group in future studies. For COPD, ambient air pollution had the worst impact on people 65–80 y of age, which refined previous research that persons >65 y of age are more sensitive to air pollution.^27^^,39^

The economic burden analysis revealed that the total economic burden due to air pollution was 486.40 and 254.74 million yuan for acute bronchitis and COPD hospitalizations, respectively, accounting for 0.015% of the regional GDP. Although the cost of an acute bronchitis hospitalization was only one-third that of a COPD hospitalization, the total economic burden of acute bronchitis hospitalization was much higher because of the large number of hospital admissions. Therefore, although existing studies around the world largely focus on COPD, asthma and pneumonia,^38,41,43,44^ more attention should be paid to acute bronchitis in future research. In terms of different pollutants, the greatest economic burden came from NO_2_, which reached 239.19 and 113.48 million yuan for acute bronchitis and COPD, respectively, followed by SO_2_, PM_10_, and PM_2.5_. This order of harmfulness was also found in previous studies in China.^29^,^33^ The results warned us that more attention should be given to NO_2_ and SO_2_. Although PM_10_ and PM_2.5_ were the leading pollutants in Chengdu, the role of NO_2_ and SO_2_ cannot be ignored when it comes to the hospitalization economic burden due to air pollution.

In subgroup analysis of gender, the results of acute bronchitis and COPD varied. The external economic burden of acute bronchitis was mostly caused by female hospital admissions, in contrast to COPD, which came mainly from males. This difference may result from the corresponding unequal gender distribution of hospital admissions. The subgroup analysis of hospital level showed that pollution-related economic burden of acute bronchitis came from primary hospitals, while that of COPD came mainly from secondary hospitals. This phenomenon may be explained by the fact that people with COPD are more inclined to go to a better hospital for treatment because COPD is more serious than acute bronchitis.

Our study has some limitations. First, we did not consider the spatial heterogeneity of air pollutants, because specific information for each hospital admission was scarce. Second, the economic burden calculated in this study included the costs due to hospitalization and absence of work but did not take into consideration costs attributed to transportation, caregiving and so on. Therefore further studies that also consider the spatial variability of air pollution and more sources of economic burden are needed.

Findings in this study will not only focus attention on environmental protection, but also provide evidence for decision makers to develop more targeted policies. Also, in the analysis of economic burden we optimized the time scale to days and considered variations in costs at different hospital levels. The results offer a more precise evaluation of the economic burden of hospitalization.

## Conclusions

Short-term exposure to NO_2_, PM_10_, PM_2.5_ and SO_2_ may contribute to increased hospital admissions of acute bronchitis and COPD, while O_3_ shows no adverse effects on these two diseases. This study indicates that these pollutants have caused a heavy economic burden of hospitalization to society. Evaluating the economic burden caused by ambient air pollutants provides a complementary approach to clarify the ill effects of air pollution. It is highly recommended that policymakers consider research conclusions when allocating health resources and formulating environmental protection policies.

## Supplementary Material

ihab071_Supplemental_File

## Data Availability

All data and material will be available from the corresponding author, Xiaoyuan Zhou upon reasonable request.

## References

[1] World Health Organization . Ambient air pollution: a global assessment of exposure and burden of disease. Geneva: World Health Organization; 2016.

[2] World Health Organization . WHO air quality guidelines for particulate matter, ozone, nitrogen dioxide and sulfur dioxide: summary of risk assessment. Geneva: World Health Organization; 2005.

[3] Liu Y , Xie S, Yu Q et al. Short-term effects of ambient air pollution on pediatric outpatient visits for respiratory diseases in Yichang city, China. Environ Pollut. 2017;227:116–24. 28458242 10.1016/j.envpol.2017.04.029

[4] Guo P , Feng W, Zheng M et al. Short-term associations of ambient air pollution and cause-specific emergency department visits in Guangzhou, China. Sci Total Environ. 2018;613–4:306–13. 10.1016/j.scitotenv.2017.09.102 28917169

[5] Castner J , Guo L, Yin Y. Ambient air pollution and emergency department visits for asthma in Erie County, New York 2007–2012. Int Arch Occup Environ Health. 2018;91(2):205–14. 29043427 10.1007/s00420-017-1270-7

[6] Zhang Y , Ni H, Bai L et al. The short-term association between air pollution and childhood asthma hospital admissions in urban areas of Hefei City in China: a time-series study. Environ Res. 2019;169:510–6. 30544078 10.1016/j.envres.2018.11.043

[7] Slama A , Śliwczyński A, Woźnica J et al. Impact of air pollution on hospital admissions with a focus on respiratory diseases: a time-series multi-city analysis. Environ Sci Pollut Res Int. 2019;26(17):16998–17009. 30929168 10.1007/s11356-019-04781-3 PMC6546668

[8] Kim SE , Honda Y, Hashizume M et al. Seasonal analysis of the short-term effects of air pollution on daily mortality in northeast Asia. Sci Total Environ. 2017;576:850–7. 27833062 10.1016/j.scitotenv.2016.10.036

[9] Dastoorpoor M , Khanjani N, Bahrampour A et al. Short-term effects of air pollution on respiratory mortality in Ahvaz, Iran. Med J Islam Repub Iran. 2018;32:30. 30159281 10.14196/mjiri.32.30 PMC6108243

[10] Maji KJ , Arora M, Dikshit AK. Burden of disease attributed to ambient PM_2.5_ and PM_10_ exposure in 190 cities in China. Environ Sci Pollut Res Int. 2017;24(12):11559–72. 28321701 10.1007/s11356-017-8575-7

[11] Chen F , Deng Z, Deng Y et al. Attributable risk of ambient PM_10_ on daily mortality and years of life lost in Chengdu, China. Sci Total Environ. 2017;581–2:426–33. 10.1016/j.scitotenv.2016.12.151 28069303

[12] Brandt S , Perez L, Kunzli N et al. Cost of near-roadway and regional air pollution-attributable childhood asthma in Los Angeles County. J Allergy Clin Immunol. 2014;134(5):1028–35. 25439228 10.1016/j.jaci.2014.09.029 PMC4257136

[13] Lu X , Lin C, Li Y et al. Assessment of health burden caused by particulate matter in southern China using high-resolution satellite observation. Environ Int. 2017;98:160–70. 27839853 10.1016/j.envint.2016.11.002

[14] Yin H , Pizzol M, Xu L. External costs of PM2.5 pollution in Beijing, China: uncertainty analysis of multiple health impacts and costs. Environ Pollut. 2017;226:356–69. 28410806 10.1016/j.envpol.2017.02.029

[15] Wan Y , Yang HW, Masui T. Health and economic impacts of air pollution in China: a comparison of the general equilibrium approach and human capital approach. Biomed Environ Sci. 2005;18(6):427–41. 16544525

[16] Maji KJ , Ye WF, Arora M et al. PM_2.5_-related health and economic loss assessment for 338 Chinese cities. Environ Int. 2018;121(Pt 1):392–403. 30245362 10.1016/j.envint.2018.09.024

[17] Franchini M , Mannucci PM, Harari S et al. The health and economic burden of air pollution. Am J Med. 2015;128(9):931–2. 25863149 10.1016/j.amjmed.2015.03.021

[18] Huang J , Pan X, Guo X et al. Health impact of China's Air Pollution Prevention and Control Action Plan: an analysis of national air quality monitoring and mortality data. Lancet Planet Health. 2018;2(7):e313–23. 30074894 10.1016/S2542-5196(18)30141-4

[19] Zhang M , Song Y, Cai X et al. Economic assessment of the health effects related to particulate matter pollution in 111 Chinese cities by using economic burden of disease analysis. J Environ Manage. 2008;88(4):947–54. 17573182 10.1016/j.jenvman.2007.04.019

[20] Babatola SS . Global burden of diseases attributable to air pollution. J Public Health Afr. 2018;9(3):813. 30687484 10.4081/jphia.2018.813 PMC6326158

[21] Huang RJ , Zhang Y, Bozzetti C et al. High secondary aerosol contribution to particulate pollution during haze events in China. Nature. 2014;514(7521):218–22. 25231863 10.1038/nature13774

[22] Qiao X , Jaffe D, Tang Y et al. Evaluation of air quality in Chengdu, Sichuan Basin, China: are China's air quality standards sufficient yet? Environ Monit Assess. 2015;187(5):250. 25877648 10.1007/s10661-015-4500-z

[23] Li L , Tan Q, Zhang Y et al. Characteristics and source apportionment of PM_2.5_ during persistent extreme haze events in Chengdu, southwest China. Environ Pollut. 2017;230:718–29. 28732335 10.1016/j.envpol.2017.07.029

[24] Tian Y , Xiang X, Juan J et al. Fine particulate air pollution and hospital visits for asthma in Beijing, China. Environ Pollut. 2017;230:227–33. 28654880 10.1016/j.envpol.2017.06.029

[25] Zhu J , Zhang X, Zhang X et al. The burden of ambient air pollution on years of life lost in Wuxi, China, 2012–2015: a time-series study using a distributed lag non-linear model. Environ Pollut. 2017;224:689–97. 28258859 10.1016/j.envpol.2017.02.053

[26] Feng W , Li H, Wang S et al. Short-term PM_10_ and emergency department admissions for selective cardiovascular and respiratory diseases in Beijing, China. Sci Total Environ. 2019;657:213–21. 30543969 10.1016/j.scitotenv.2018.12.066

[27] Tao Y , Mi S, Zhou S et al. Air pollution and hospital admissions for respiratory diseases in Lanzhou, China. Environ Pollut. 2014;185:196–201. 24286694 10.1016/j.envpol.2013.10.035

[28] Wang C , Feng L, Chen K. The impact of ambient particulate matter on hospital outpatient visits for respiratory and circulatory system disease in an urban Chinese population. Sci Total Environ. 2019;666:672–9. 30812001 10.1016/j.scitotenv.2019.02.256

[29] Tian Y , Liu H, Liang T et al. Ambient air pollution and daily hospital admissions: a nationwide study in 218 Chinese cities. Environ Pollut. 2018;242(Pt B):1042–9. 30096542 10.1016/j.envpol.2018.07.116

[30] Chen R , Chu C, Tan J et al. Ambient air pollution and hospital admission in Shanghai, China. J Hazard Mater. 2010;181(1–3):234–40. 20537796 10.1016/j.jhazmat.2010.05.002

[31] Jo C. Cost-of-illness studies: concepts, scopes, and methods. Clin Mol Hepatol. 2014;20(4):327–37. 25548737 10.3350/cmh.2014.20.4.327 PMC4278062

[32] Esposito S , Tenconi R, Lelii M et al. Possible molecular mechanisms linking air pollution and asthma in children. BMC Pulm Med. 2014;14:31. 24581224 10.1186/1471-2466-14-31 PMC3941253

[33] Gao N , Li C, Ji J et al. Short-term effects of ambient air pollution on chronic obstructive pulmonary disease admissions in Beijing, China (2013–2017). Int J Chron Obstruct Pulmon Dis. 2019;14:297–309. 30774327 10.2147/COPD.S188900 PMC6350834

[34] Luong LMT , Phung D, Dang TN et al. Seasonal association between ambient ozone and hospital admission for respiratory diseases in Hanoi, Vietnam. PLoS One. 2018;13(9):e0203751. 30248114 10.1371/journal.pone.0203751 PMC6152873

[35] Chen K , Glonek G, Hansen A et al. The effects of air pollution on asthma hospital admissions in Adelaide, South Australia, 2003–2013: time-series and case-crossover analyses. Clin Exp Allergy. 2016;46(11):1416–30. 27513706 10.1111/cea.12795

[36] Karakatsani A , Samoli E, Rodopoulou S et al. Weekly personal ozone exposure and respiratory health in a panel of Greek schoolchildren. Environ Health Perspect. 2017;125(7):077016. 28749779 10.1289/EHP635 PMC5744680

[37] O'Lenick CR , Chang HH, Kramer MR et al. Ozone and childhood respiratory disease in three US cities: evaluation of effect measure modification by neighborhood socioeconomic status using a Bayesian hierarchical approach. Environ Health. 2017;16:36. 28381221 10.1186/s12940-017-0244-2 PMC5382444

[38] Zhang Z , Hong Y, Liu N. Association of ambient particulate matter 2.5 with intensive care unit admission due to pneumonia: a distributed lag non-linear model. Sci Rep. 2017;7:8679. 28819316 10.1038/s41598-017-08984-x PMC5561234

[39] Capraz O , Deniz A, Dogan N. Effects of air pollution on respiratory hospital admissions in Istanbul, Turkey, 2013 to 2015. Chemosphere. 2017;181:544–50. 28463729 10.1016/j.chemosphere.2017.04.105

[40] Phung D , Hien TT, Linh HN et al. Air pollution and risk of respiratory and cardiovascular hospitalizations in the most populous city in Vietnam. Sci Total Environ. 2016;557–8:322–30. 10.1016/j.scitotenv.2016.03.070 27016680

[41] Qiu H , Yu H, Wang L et al. The burden of overall and cause-specific respiratory morbidity due to ambient air pollution in Sichuan Basin, China: a multi-city time-series analysis. Environ Res. 2018;167:428–36. 30121467 10.1016/j.envres.2018.08.011

[42] Muñoz F , Carvalho MS. [Effect of exposure time to PM_10_ on emergency admissions for acute bronchitis]. Cad Saúde Púb. 2009;25(3):529–39. 10.1590/s0102-311x2009000300008 19300842

[43] Qiu H , Tan K, Long F et al. The burden of COPD morbidity attributable to the interaction between ambient air pollution and temperature in Chengdu, China. Int J Environ Res Public Health. 2018;15(3):492. 10.3390/ijerph15030492 PMC5877037 29534476

[44] Wang C , Xu J, Yang L et al. Prevalence and risk factors of chronic obstructive pulmonary disease in China (the China Pulmonary Health [CPH] study): a national cross-sectional study. Lancet. 2018;391(10131):1706–17. 29650248 10.1016/S0140-6736(18)30841-9

