# Supplementary material for: Pricing air pollution: evidence from short-term exposure to air pollution on hospitalization of acute bronchitis and chronic obstructive pulmonary disease in southwestern China
Source: Int Health. 2021 Nov 19;14(6):572–9. doi: 10.1093/inthealth/ihab071 (PMC9623494; doi:10.1093/inthealth/ihab071)
Supplement: ihab071_Supplemental_File [file ihab071_supplemental_file.doc]

# Supplementary Table

## Supplementary Table S1. Percentage changes of hospital admission with a 10 µg/m^3^ increase in pollutant concentrations for different degree of freedom(df) of temperature and long-term trend.

| Disease | df of temperature | df of long-term trend | Percentage changes of hospital admission (%) | | | | |
| --- | --- | --- | --- | --- | --- | --- | --- |
|  |  |  | NO_2_ | O_3_ | PM_10_ | PM_2.5_ | SO_2_ |
| Acute  bronchitis | 2 | 6 | 2.10(1.54,2.67) | -0.23(-0.56,0.10) | 1.72(0.70,2.76) | 1.11(0.19,2.04) | 3.63(2.49,4.78) |
|  | 3 | 6 | 2.11(1.54,2.68) | -0.23(-0.56,0.10) | 1.75(0.72,2.79) | 1.13(0.21,2.06) | 3.69(2.55,4.85) |
|  | 4 | 6 | 2.12(1.55,2.69) | -0.23(-0.56,0.10) | 1.78(0.75,2.82) | 1.14(0.22,2.07) | 3.70(2.56,4.86) |
|  | 2 | 7 | 1.93(1.38,2.49) | -0.25(-0.57,0.08) | 1.56(0.55,2.57) | 1.01(0.10,1.92) | 3.19(2.07,4.31) |
|  | 3 | 7 | 1.95(1.39,2.51) | -0.25(-0.58,0.08) | 1.58(0.57,2.59) | 1.00(0.10,1.92) | 3.28(2.17,4.41) |
|  | 4 | 7 | 1.95(1.40,2.51) | -0.25(-0.58,0.08) | 1.60(0.59,2.61) | 1.01(0.11,1.92) | 3.29(2.17,4.42) |
|  | 2 | 8 | 1.88(1.33,2.43) | -0.21(-0.53,0.12) | 1.51(0.52,2.52) | 1.06(0.16,1.96) | 3.11(2.00,4.23) |
|  | 3 | 8 | 1.89(1.34,2.44) | -0.21(-0.53,0.12) | 1.52(0.53,2.53) | 1.05(0.15,1.96) | 3.17(2.06,4.29) |
|  | 4 | 8 | 1.90(1.35,2.45) | -0.22(-0.54,0.11) | 1.54(0.54,2.54) | 1.05(0.16,1.96) | 3.19(2.08,4.31) |
| COPD | 2 | 6 | 2.36(1.58,3.15) | 0.28(-0.18,0.74) | 2.46(1.11,3.82) | 1.81(0.60,3.04) | 3.88(2.37,5.41) |
|  | 3 | 6 | 2.34(1.56,3.13) | 0.29(-0.17,0.75) | 2.40(1.05,3.76) | 1.75(0.55,2.98) | 3.78(2.27,5.31) |
|  | 4 | 6 | 2.34(1.56,3.13) | 0.29(-0.17,0.75) | 2.41(1.06,3.78) | 1.76(0.55,2.98) | 3.78(2.27,5.31) |
|  | 2 | 7 | 2.26(1.48,3.04) | 0.30(-0.16,0.77) | 2.31(0.97,3.67) | 1.71(0.50,2.93) | 3.59(2.10,5.11) |
|  | 3 | 7 | 2.24(1.46,3.02) | 0.30(-0.16,0.76) | 2.29(0.95,3.65) | 1.71(0.50,2.93) | 3.51(2.01,5.03) |
|  | 4 | 7 | 2.24(1.46,3.02) | 0.30(-0.16,0.77) | 2.29(0.95,3.65) | 1.71(0.50,2.93) | 3.51(2.01,5.03) |
|  | 2 | 8 | 2.21(1.44,2.99) | 0.33(-0.12,0.79) | 2.29(0.96,3.65) | 1.70(0.49,2.92) | 3.53(2.04,5.04) |
|  | 3 | 8 | 2.20(1.42,2.98) | 0.33(-0.13,0.79) | 2.28(0.94,3.63) | 1.72(0.51,2.94) | 3.45(1.96,4.97) |
|  | 4 | 8 | 2.20(1.42,2.98) | 0.33(-0.13,0.78) | 2.28(0.95,3.64) | 1.72(0.51,2.94) | 3.45(1.96,4.97) |

# Supplementary Figures


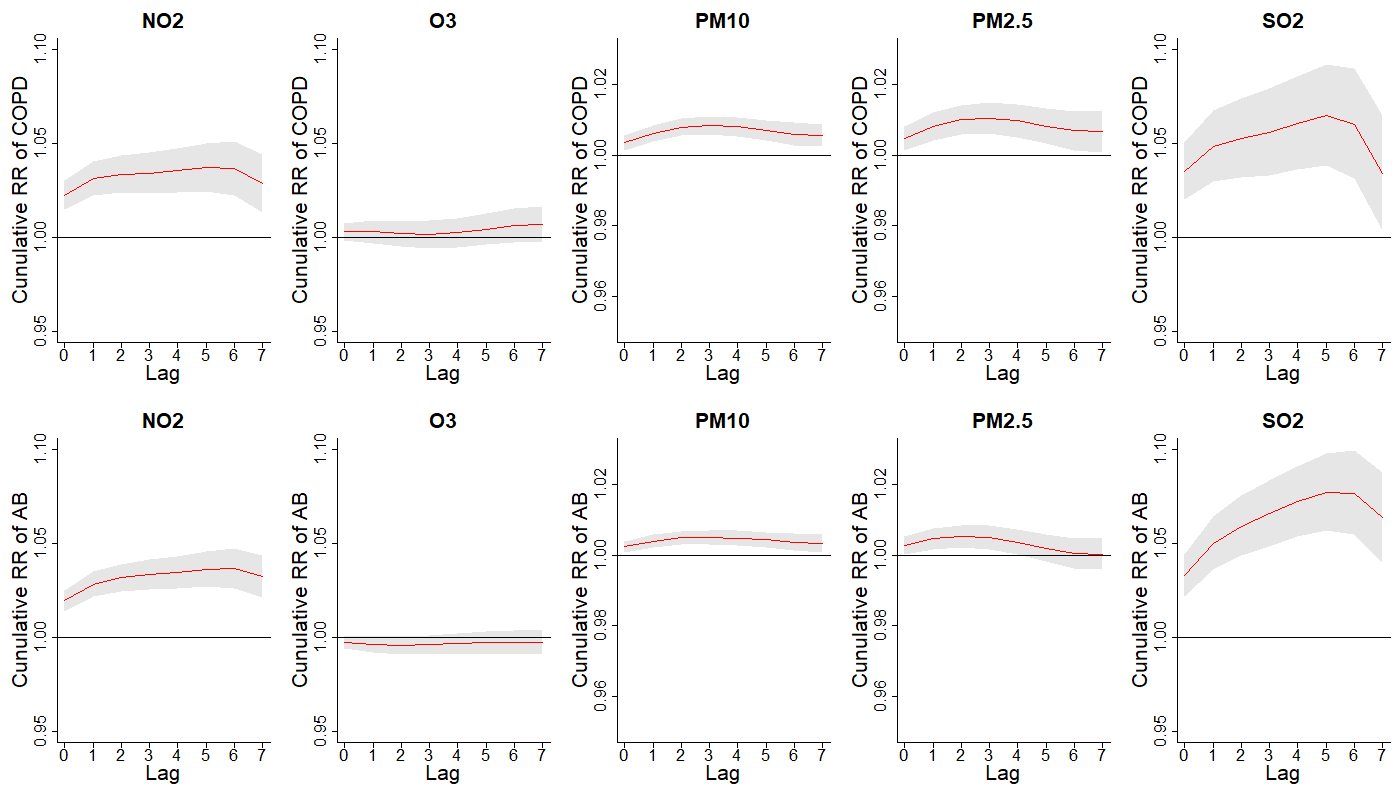


## Supplementary Figure S1. Overall cumulative exposure-response associations between ambient air pollutants and hospital admissions.


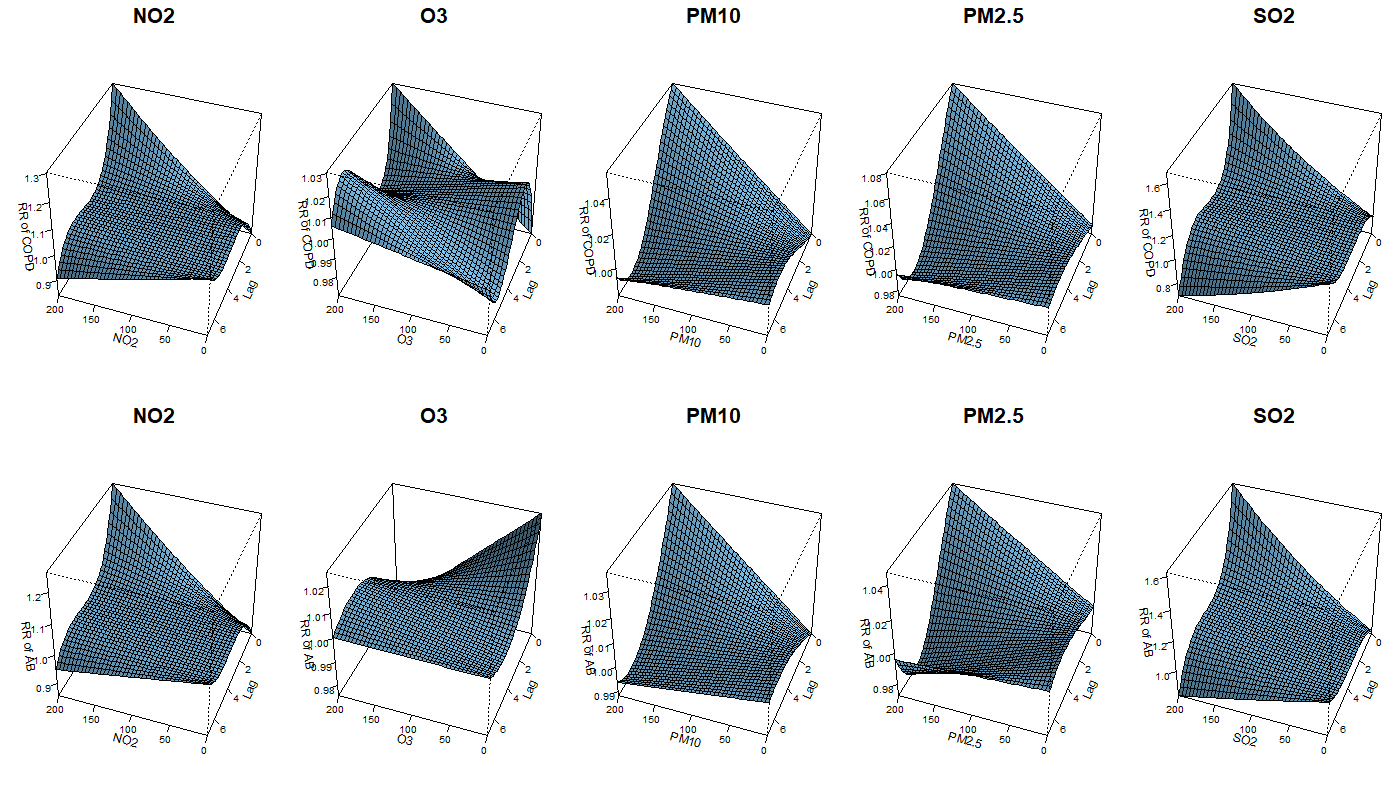


## Supplementary Figure S2. 3-D graphs of bi-dimensional exposure-lag response surfaces of ambient air pollutants on hospital admissions at different lag days.


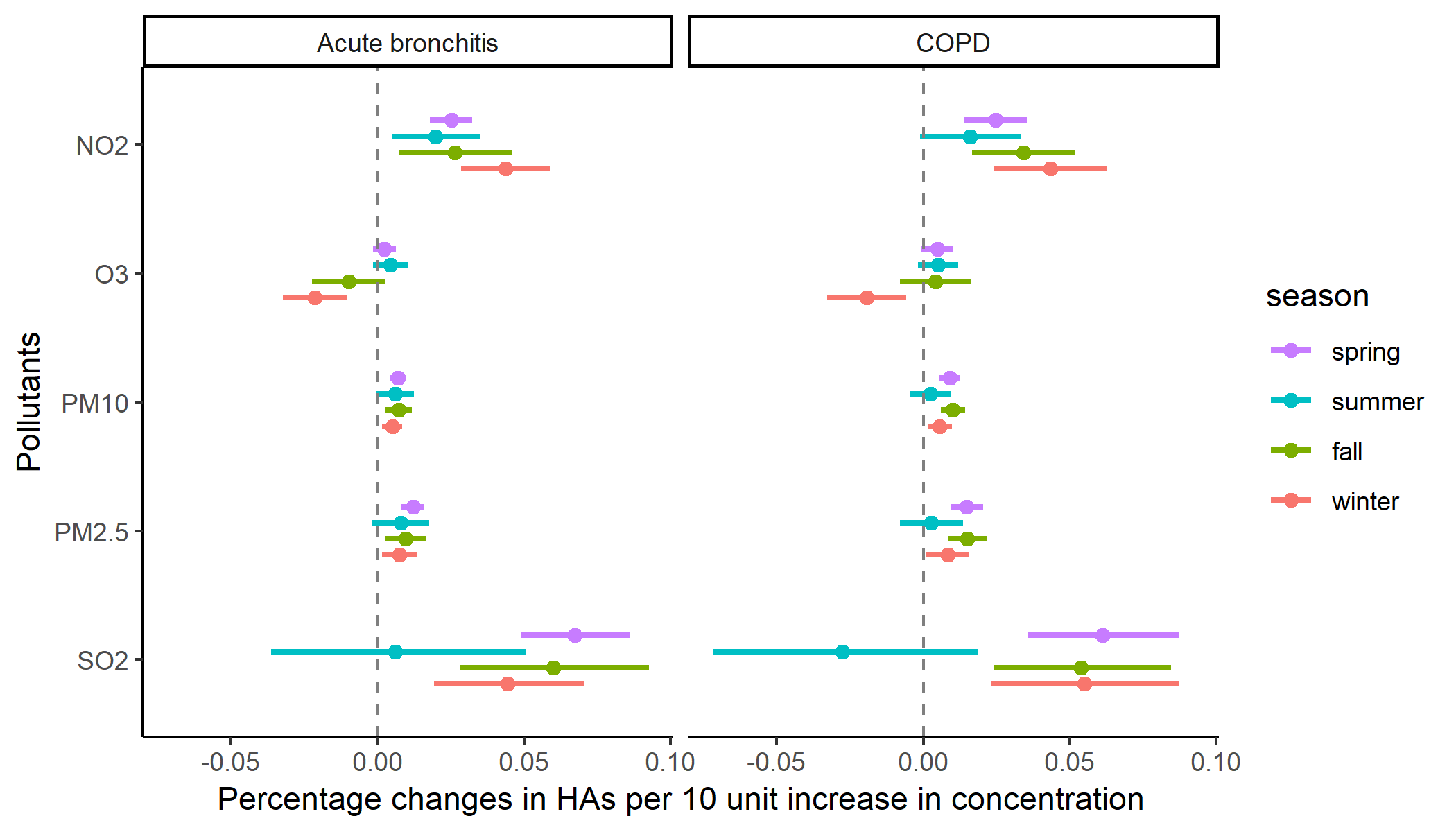


## Supplementary Figure S3. Percentage changes of hospital admission with a 10 µg/m^3^ increase in pollutant concentrations in different seasons.
